# Assessment of dose perturbations for metal stent in photon and proton radiotherapy plans for hepatocellular carcinoma

**DOI:** 10.1186/s13014-022-02100-8

**Published:** 2022-07-16

**Authors:** Boram Lee, Sungkoo Cho, Hee Chul Park, Sang-Won Kang, Jae-Sung Kim, Jin-Beom Chung

**Affiliations:** 1 Department of Radiation Oncology, Seoul National University Bundang Hospital, 82 Gumi-ro 173 Beon-gil, Bundang-gu, Seongnam-si, Gyeonggi-do 13620, Seongnam, Korea; 2 Department of Radiation Oncology, Samsung Medical Center, Seoul, South Korea; 3 Department of Radiation Oncology, Samsung Medical Center, Sungkyunkwan University School of Medicine, Seoul, South Korea

**Keywords:** Metal stent, Proton, Photon, Monte Carlo simulation, Hepatocellular carcinoma

## Abstract

**Background:**

The present study aimed to investigate the dosimetric impact of metal stent for photon and proton treatment plans in hepatocellular carcinoma.

**Methods:**

With computed tomography data of a water-equivalent solid phantom, dose perturbation caused by a metal stent included in the photon and proton treatment of hepatocellular carcinoma was evaluated by comparing Eclipse and RayStation treatment planning system (TPS) to a Monte Carlo (MC) based dose calculator. Photon and proton plans were created with anterior–posterior/posterior-anterior (AP/PA) fields using a 6 MV beam and AP/PA fields of a wobbling beam using 150 MeV and a 10 cm ridge filter. The difference in dose distributions and dosimetric parameters were compared depending on the stent's positions (the bile duct (GB) and intestinal tract (GI)) and angles (0°, 45°, and 90°). Additionally, the dose variation in the target volume including the stent was comparatively evaluated through dose volume histogram (DVH) analysis. And the comparison of clinical cases was carried out in the same way.

**Results:**

Percentage differences in the dosimetric parameters calculated by MC ranged from − 7.0 to 3.9% for the photon plan and − 33.7 to 4.3% for the proton plan, depending on the angle at which the GB and GI stents were placed, compared to those without the stent. The maximum difference was observed at the minimum dose (D_min_), which was observed in both photon and proton plans in the GB and GI stents deployed at a 90° incidence angle. The parameter differences were greater in the proton plan than in photon plan. The target volume showed various dose variations depending on positions and angles of stent for both beams. Compared with no-stent, the doses within the target volume containing the GI and GB stents for the photon beam were overestimated in the high-dose area at 0°, nearly equal within 1% at 45°, and underestimated at 90°. These doses to the proton beam were underestimated at all angles, and the amount of underdose to the target volume increased with an increase in the stent angle. However, the difference was significantly greater with the proton plan than the photon plan.

**Conclusions:**

Dose perturbations within the target volume due to the presence of the metal stent were not observed in the TPS calculations for photon and proton beams, but MC was used to confirm that there are dose variations within the target volume. The MC results found that delivery of the treatment beam avoiding the stent was the best method to prevent target volume underdose.

## Background

Hepatocellular carcinoma (HCC), a malignant tumour of the liver, is one of the most difficult cancers to detect and has a poor prognosis. Depending on the patient's condition, it is treated with surgical resection, radiofrequency ablation, transarterial chemoembolization (TACE), and radiotherapy (RT) [1–3]. Among these treatments, RT has not been widely used for HCC in the past due to the high resistance of HCC tissue/tumour cells to radiation, the dose error occurring in moving organs, and the side effects caused by radiation [4]. However, because of an improvement in RT technology and clinical experience, the adaptation of RT for the treatment of HCC has been increasing. Moreover, the use of volumetric modulation arc therapy (VMAT), tomotherapy, and proton beam therapy (PBT) has been increasing recently [1, 5, 6].

HCC patients are treated with VMAT or PBT, depending on the particular case [5]. Few of the patients treated with either of these methods are treated with a stent inserted into the body during RT. The stent is generally used for bile duct dilatation, portal vein, and duodenal stenosis among the treatment sites, and the type and silicone coating are determined depending on the treatment site and the condition of patient. Stents made from a variety of materials, including nickel, titanium, iron, hafnium, copper, boron, and niobium, and using high atomic number (High-Z) materials can affect the amount of radiation delivered to the surrounding tissue [7]. Self-expanding metal stents (SEMS), which are usually applied to the treatment of disease-induced stenosis, have the advantage of acting as fiducial markers that increase the accuracy of high-dose treatments [8]. Because this type of stent is not a high-Z material such as gold, it does not generate strong artefacts [9]. Fiducial markers that enable accurate positioning have an advantage in reducing the target margin [10]. However, if the stent is placed in the pathway of the radiation field, it causes dose perturbations [8, 11–13]. The dose perturbation for the photon beam is primarily due to secondary electrons and scattering caused by the metallic components within the stent [7]. Metal stents developed using nitinol, an alloy of nickel and titanium, have been clinically tested and are the most widely used [7, 8, 10, 13, 14]. Especially in proton therapy, the dose perturbations caused by metal stents will result in an underdose and overdose in that part of the target volume containing the metal stent due to secondary electrons and multi-scattering. Therefore, the range uncertainty requires additional margins when creating a treatment plan. Currently, a method used mainly in the treatment of patients with SEMS in clinical practice is to override the stent to the surrounding tissue. It is very difficult to accurately calculate the effect of the stent on the dose distribution with a treatment planning system (TPS). The reasons are as follows. First, it is difficult to define a physical area affected by the stent. The minimum voxel size reconstruction by computed tomography (CT) is approximately 1 mm^3^; however, the thickness of the stent used in clinic is 0.16 mm, which is relatively small, resulting in image artefacts such as blurring [10]. In addition, the TPSs that are used possess a limited ability to define regions of interest (ROIs), making it impossible to define a small stent volume. Second, towing to limitations of the TPSs, the reconstructed physical density of the stent voxel is averaged with the surrounding values and thus is evaluated to be lower than the density of the actual stent. To eliminate these problems, clinics mainly perform dose calculations by replacing the density of the stent with the density of the surrounding tissue. However, it is necessary to accurately recognise the effect of the dose perturbation caused by the stent. Several previous studies have been conducted to verify the effect of metal stents on the dose [7, 11, 12, 15]. The effects of the stents were mainly studied for RT using a photon beam. In RT using a proton beam, the effects were evaluated through Monte Carlo (MC) simulations and through measurements using films. These studies were performed to verify the range of the differences in a specific area [16]. Only a few studies focused on the dosimetric evaluation of the effects that occur in the volume where the stent is inserted.

This study aimed to understand the volumetric effect of dose perturbations induced during RT using photon and proton beams, by metal stents used in HCC patients. The dose coverage of the target volume was evaluated at the location of the stent (GB and GI stents) according to different beam incident directions (0°, 45°, and 90°), and the effect of dose perturbation by the stent was evaluated in clinical practice.

## Methods

**Fig. 1:**
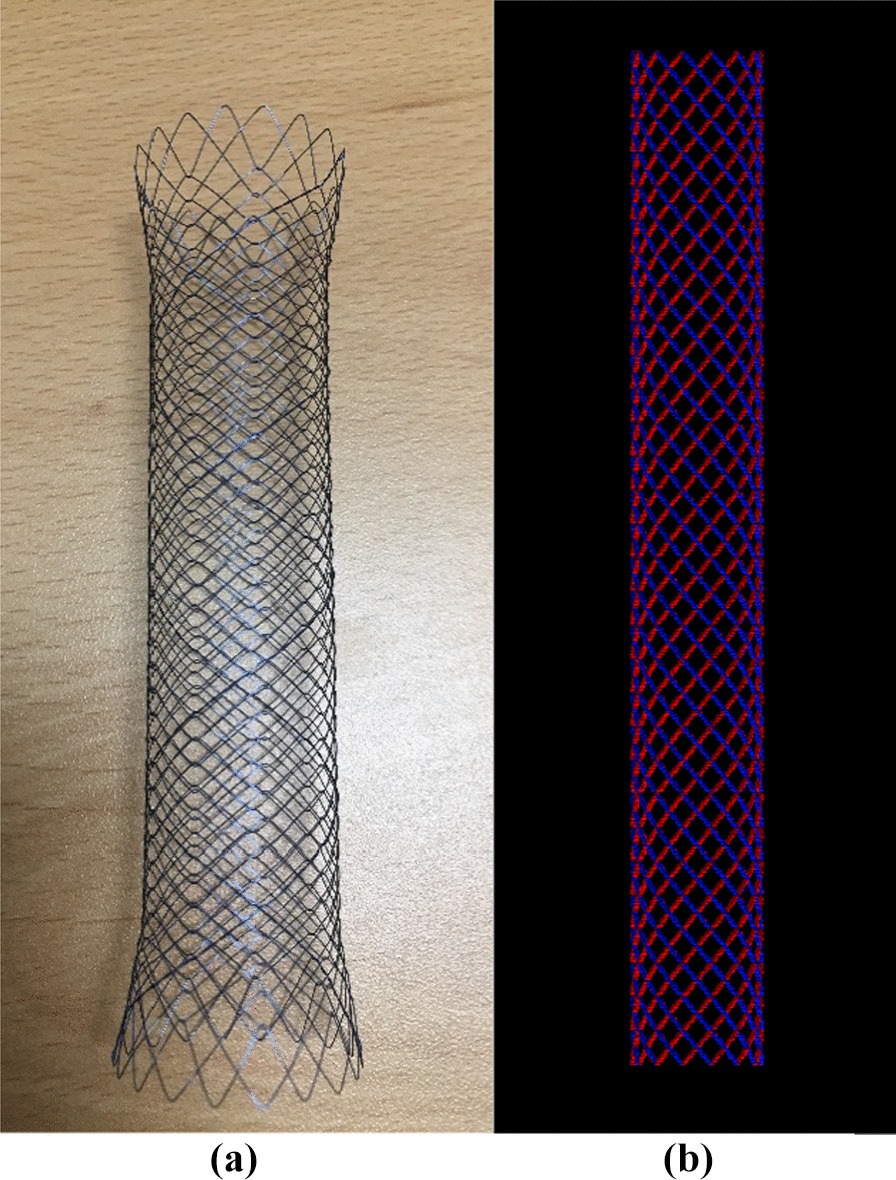
Metal stent used in clinical practice **a** and metal stent modeled by computer **b** for dose calculation

In this study, dose perturbation caused by metal stents was evaluated using MC simulations and TPSs for photon and proton beams. For the evaluation of dose calculation with TPS, CT data were obtained for 1.25 mm thick slices in a water-equivalent solid phantom containing a metal stent. Subsequently, MC simulations were performed to evaluate the dose perturbation due to the metal stent in three dimensions [8]. Geant4 (v10.3) and GATE (v8.1), which have the advantages of simple geometric configuration and fast computation time, were used for the MC configuration. Nitinol, an alloy of nickel and titanium currently used in clinical practice, was used for the metal stent. Component data for the metal stents were provided by the manufacturer. The metal stent used in clinical practice and the modelled stent used in the MC simulation for dose calculation are shown in Fig. 1. The stent used for evaluating the dose was constructed as follows. The mass density of the nitinol was 6.8 g/cm^3^. Two types of stents were used to determine the effect of the stent size: one in the bile duct (the GB stent) and another in the gastrointestinal tract (the GI stent). The diameter of the metal wire constituting the metal stent was 0.16 mm; when constructed in cross-section, the stent consisted of 28 wires for the bile duct and 36 wires for the gastrointestinal tract. The material composition was the same for the bile duct and gastrointestinal tract, and the diameter of each stent was between 1 and 2 cm when not inserted into the body.

A Truebeam (Varian Medical Systems) linear accelerator and a proton therapy system (Sumitomo Heavy Industries Ltd.) were used to study the dose difference caused by metal stents in a photon beam and proton beam, respectively. The virtual machine system completed in previous studies [17–19] was used as the equipment modelling step of the MC calculation. We checked the percent depth dose and dose profile consistency for various energies to validate the MC of the equipment in the previous studies, and used the phase space file that were formatted according to recommendations of the International Atomic Energy Agency (IAEA) that confirmed the agreement with a mean error of maximum 0.5%. Moreover, a compact cluster was designed for MC calculation [20].

**Fig. 2:**
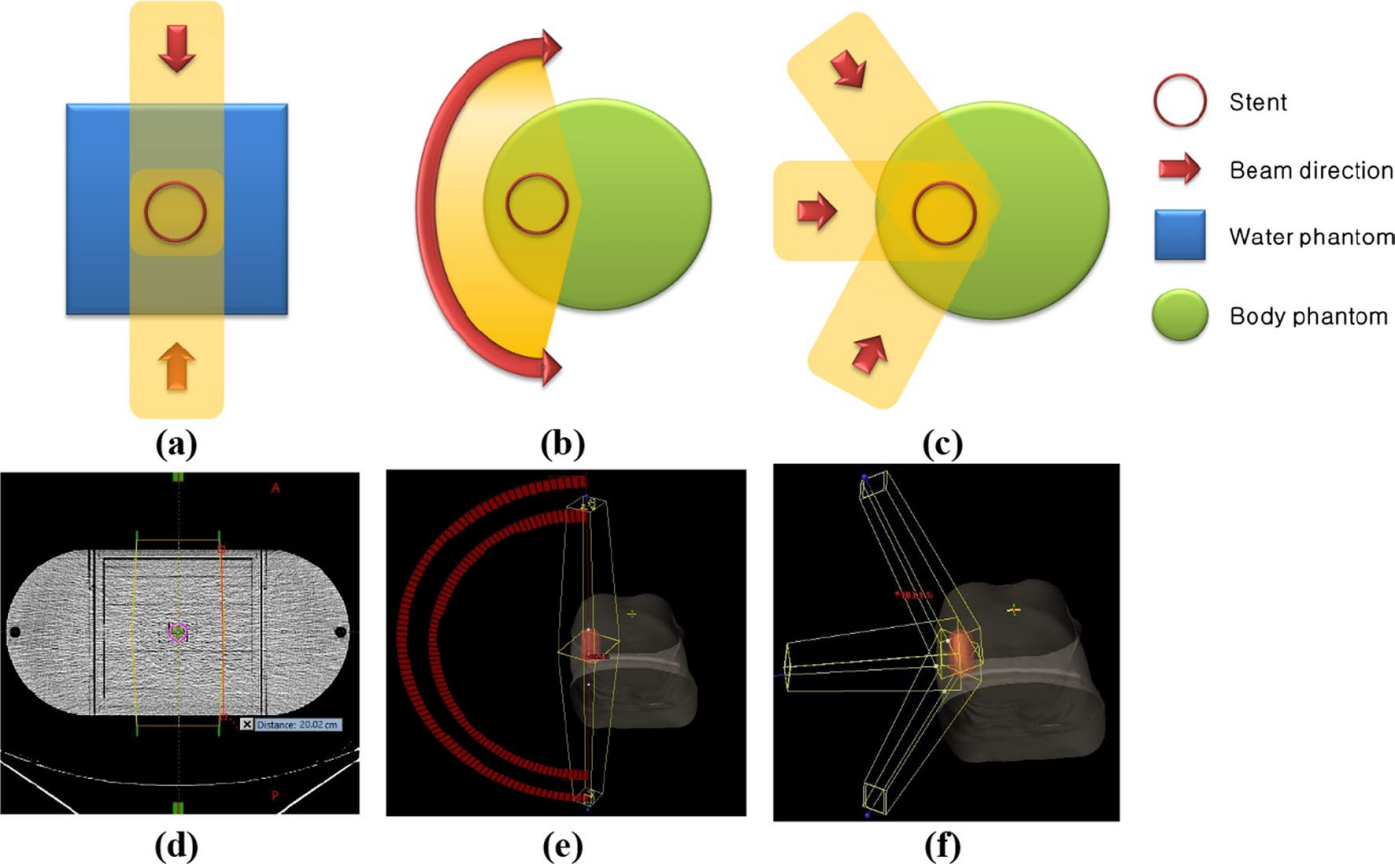
Schematic and beam configuration of AP/PA fields **a**, **d**, VMAT **b**, **e**, and double scattering **c**, **f** plans to evaluate the dose perturbation by the metal stent

Figure 2 shows the schematic and beam configuration of the anterior–posterior/posterior-anterior (AP/PA) field technique and VMAT, and double scattering plans for evaluating dose perturbation by metal stents. As shown in Fig. 2, the photon plans were generated with Eclipse TPS using Acuros XB algorithm (v 16.7, Varian Medical Systems) for the AP/PA fields (Fig. 2a, d) and VMAT (Fig. 2b, e) techniques using a 6 MV beam, and the proton plans were created with Raystation TPS using pencil beam algorithm (PBv4.2, RaySearch Laboratories) for AP/PA fields (Fig. 2a, d) and three-field double scattering of the wobbling beam (Fig. 2c, f) using a 150 MeV energy and 10 cm ridge filter [21]. The plans for the photon and proton beams were both created under the same conditions in the TPS and MC simulation. In the AP/PA plan, the field of each beam was opened with a margin of 5 mm to sufficiently cover that area based on the size of the stent to evaluate the dose in a stable area. A water phantom of 20 cm × 20 cm was constructed, and a metal stent was placed in the centre of the phantom. The effect of various beam angles on the stent inserted into the patient was considered. The stent was fixed at angles of 0°, 45°, and 90°, respectively, to evaluate the angular dependence by calculating the dosimetric difference depending on the incident angle of the beam [8]. In addition, to evaluate the effect of dose perturbation by the stent in actual clinical practice, the dose perturbation was evaluated by virtualizing digital human phantom together with the stent [22]. The human phantom data was converted into metadata format and the dose was calculated with the MC engine through stoichiometric correction [22]. In the case of photon beam, the two-half arc technique with one isocentre was used as the VMAT most used in clinical practice, and in the case of proton beam, the three-field technique was planned using the double scattering method. The dose perturbation according to the stent was evaluated and compared using the constructed treatment plan.

The range cut value of the water phantom was 0.01 mm with respect to the size of the wire, and the dose value calculated by the DoseActor function was reconstructed into a voxel size of 1 mm^3^. The physics list library for the calculation of the photon and proton beams used the QuarkGluonStrongG4Precompound-BInaryCascade-HighPrecision neutron-ElectroMagnetic opt Z (QGSP-BIC-HP-EMZ) reference physics list [23].

The calculated results were sent to a system that had been programmed to be converted into a digital imaging and communications in medicine (DICOM) format compatible with the TPS. Because the digital value in the uncorrected simulation required a tool for analysis, we set the configuration to import the frame ID value into the TPS and performed the dose analysis using the TPS function. The output values calculated in the MC simulation were compared and analysed as relative values. For evaluation, a contour was made for dose analysis by expanding 5 mm, based on the metal stent.

For AP/PA field technique, VMAT, and double scattering plans, dosimetric parameters such as the maximum dose (D_max_), minimum dose (D_min_), mean dose (D_mean_), and dose of 1% volume (D_1%_) for target volume were evaluated to investigate the effect in the coverage of target volume due to the metal stent. In addition, the difference in dose volume histograms (DVHs) were compared according to the type of the metal stent for the photon and proton beams.

## Results

**Fig. 3:**
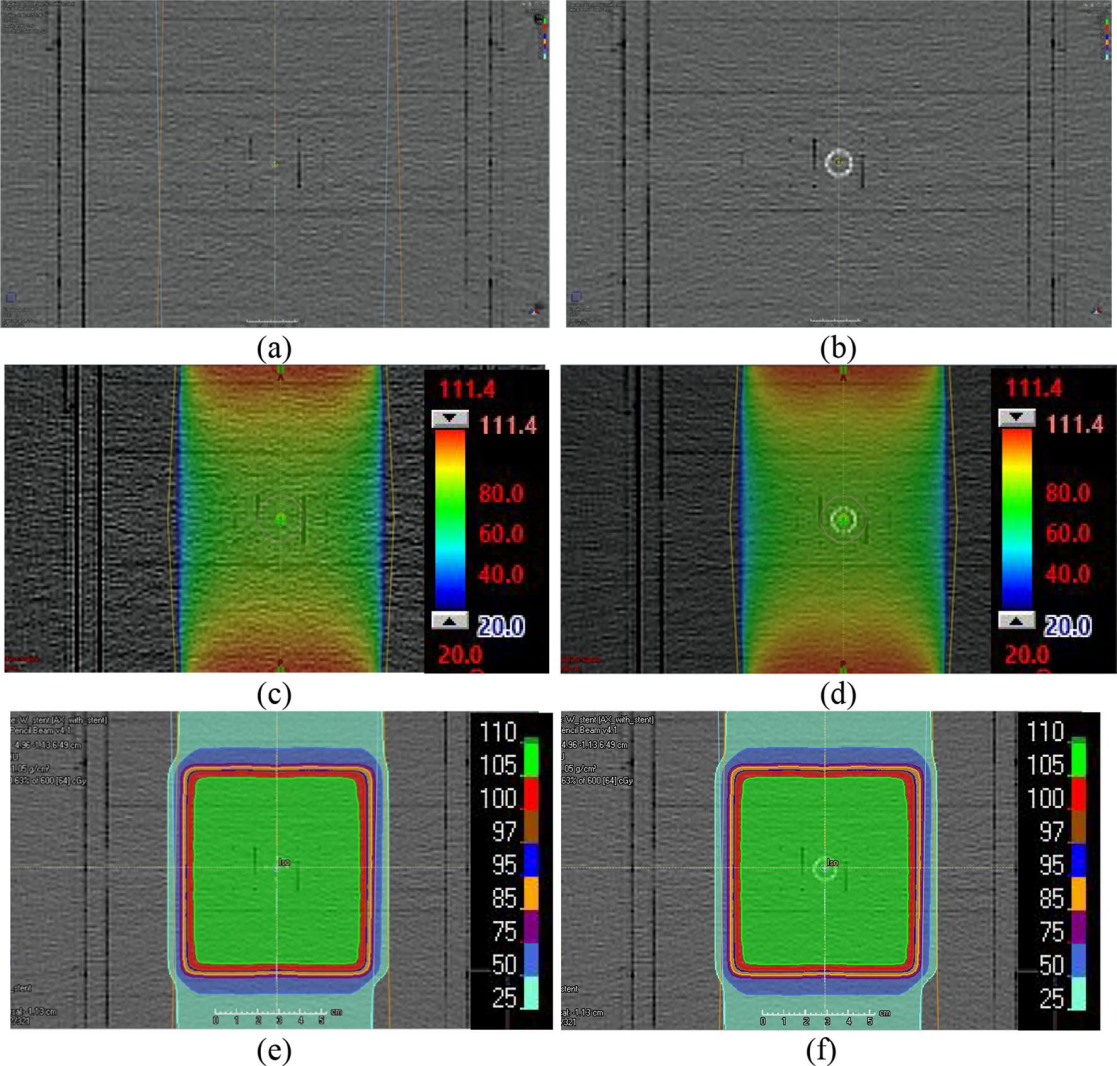
Scanned phantom image **a** without the metal stent and dose distributions calculated from TPSs for photon **c** and proton **e** plans, and scanned phantom image **b** with the metal stent and dose distributions calculated from TPSs for photon **d** and proton **e** plans

**Fig. 4:**
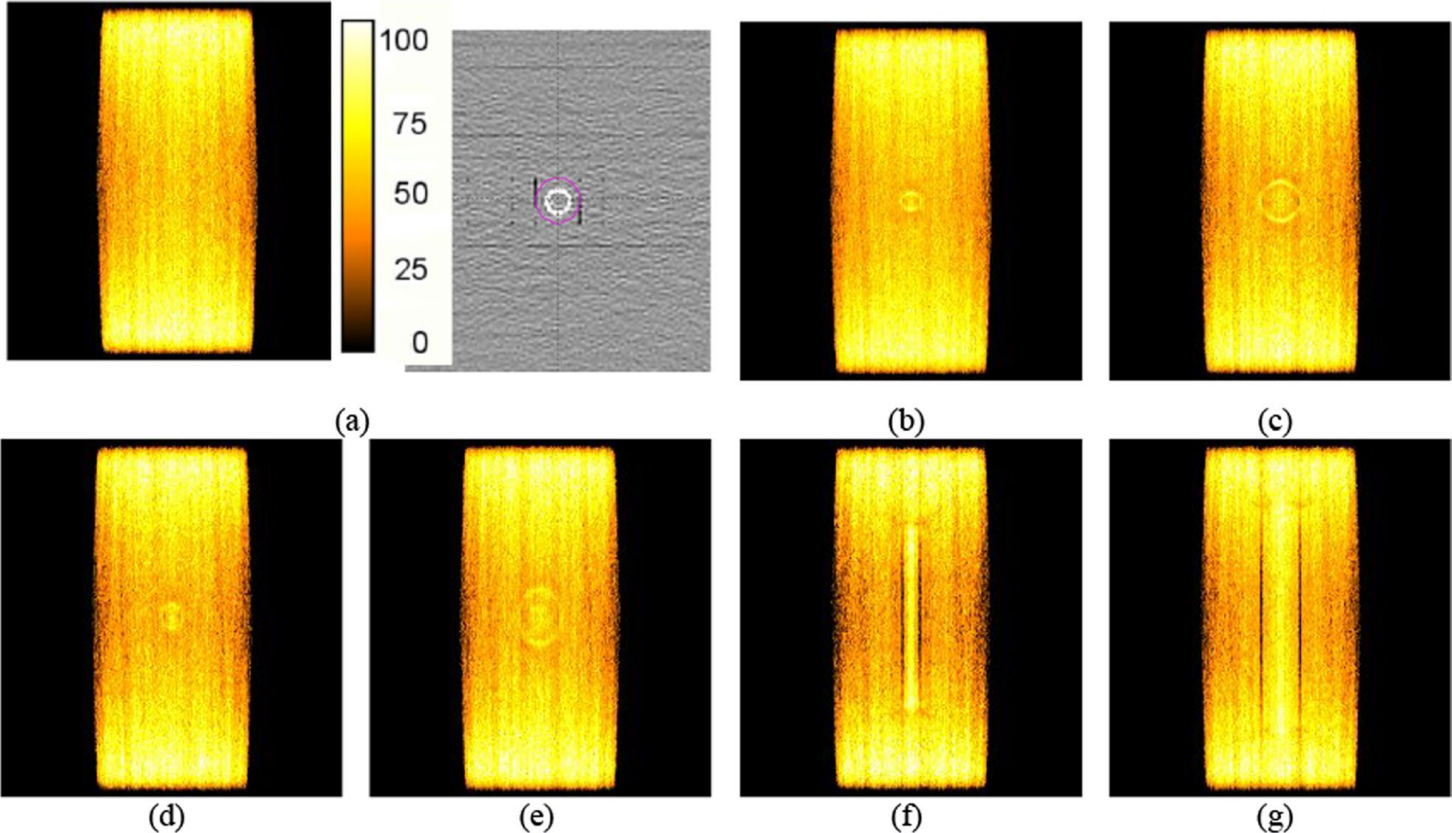
Axial dose distributions calculated from MC simulation for the photon beam. Dose distribution without the metal stent **a**, dose distributions calculated from the GB stent **b** and GI stent **c** placed at an angle of 0°, dose distributions calculated from the GB stent **d** and GI stent **e** placed at angle of 45°, and dose distributions calculated for the GB stent **f** and GI stent **g** placed at an angle of 90°

**Fig. 5:**
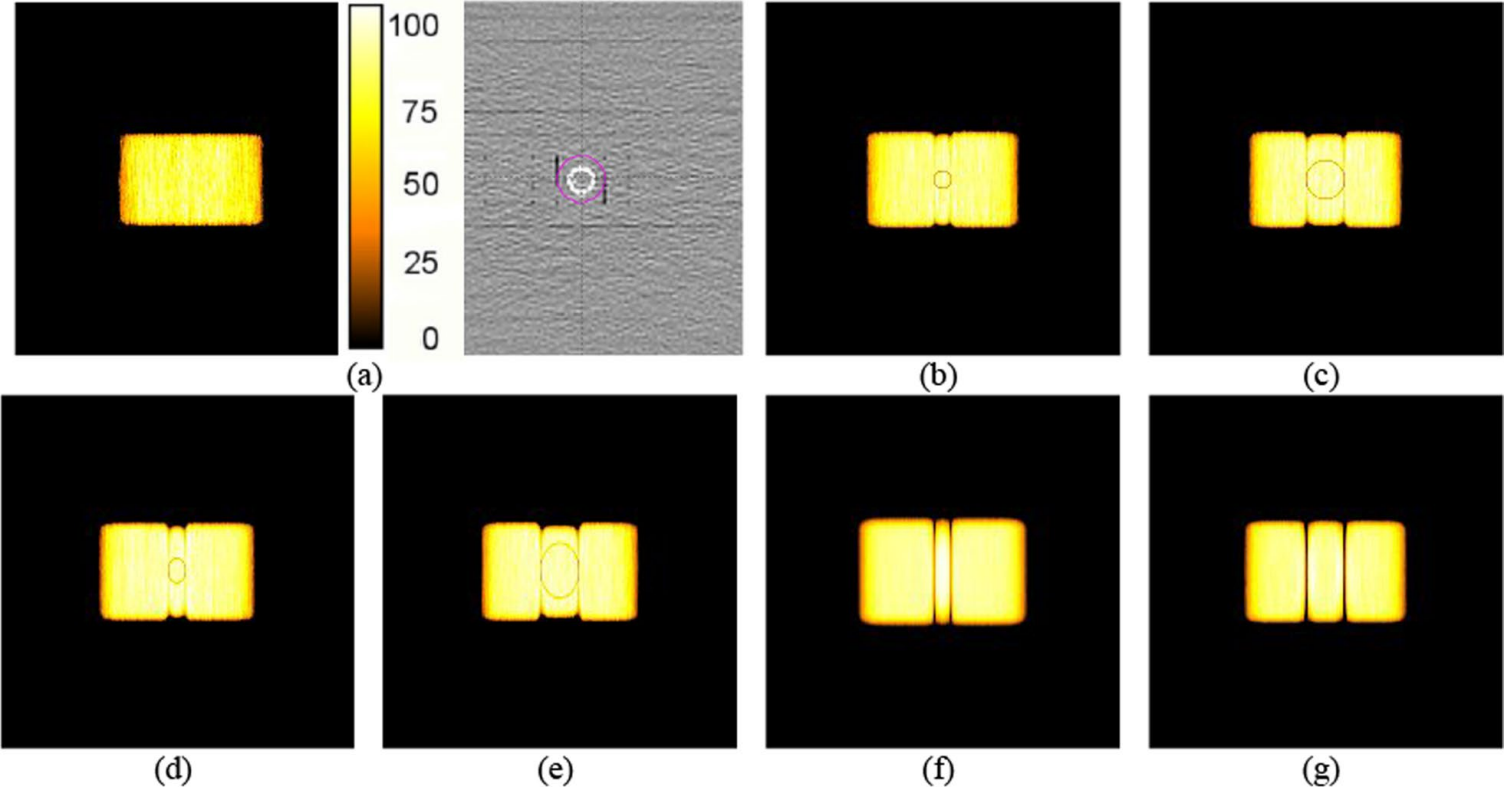
Axial dose distributions calculated from MC simulation for the proton beam. Dose distribution without the metal stent **a**, dose distributions calculated from the GB stent **b** and GI stent **c** placed at an angle of 0°, dose distributions calculated from the GB stent **d** and GI stent **e** placed at angle of 45°, and dose distributions calculated for the GB stent **f** and GI stent **g** placed at an angle of 90°

The dose distributions calculated from TPSs for the photon and proton AP/PA fields using phantom images with and without metal stents are shown in Fig. 3. As shown in the figure, there were no significant dose perturbations in the area containing the metal stent in the photon and proton plans obtained from the TPS. This means that TPS calculations for photon and proton beams did not accurately calculate the dose perturbation in the area containing the metal stent. The differences between the axial dose distributions calculated by the MC simulations for the photon and proton AP/PA fields, with and without the stent, are shown in Figs. 4 and 5. As shown in these figures, it was visually confirmed that the dose was changed compared to the dose distribution calculated without the stent due to the change in the stent size depending on the various position angles (0°, 45°, and 90°) of the stent.

The differences in dosimetric parameters on photon and proton plans calculated using TPS and MC simulation for GI and GB stents according to the incident beam angle are listed in Tables 1 and 2 to 4 compared to the those without the stent. Table 1 shows that the various dosimetric parameters of TPS plans using photon and proton beams are within a 0.5% difference with and without GB and GI stents. Tables 2 and 3 show the percentage difference in dosimetric parameters according to the incident beam angle of MC simulations with GB and GI stents compared to MC simulations without stent for photon and proton beams. In the MC simulation for the photon beam, the percentage difference in dosimetric parameters when the stent was inserted into the body was − 7 to 3.9% compared to the case without the stent. The maximum percentage difference in D_1%_ was observed 4.0% overdose in the GB stent positioned at 0°. The differences of D_min_ were within 1.1% of the no-stent value for the GB stent and the GI stent when the stents were placed at 0° and 45°. The D_min_ value obtained at an angle of 90° showed a relatively large difference, with a decrease of 5.9% for the GB stent and 7.0% for the GI stent, respectively. The values of D_mean_ were not significantly different for the stents positioned at 0° and at 45°. For the stents positioned at 90°, there were small differences for both the GB stent (− 1.7%) and GI stent (0.7%). The differences in the dosimetric parameters (compared to not using the stent) were large for both the GB stent and the GI stent located at 90°.

**Table 1:** Percentage differences in dosimetric parameters on TPS plans with GB and GI stents compared to the TPS plans without stent for photon and proton beams

	Photon		Proton	
	GB stent	GI stent	GB stent	GI stent
D_max_ (%)	0.5	0.2	0.4	0.3
D_min_ (%)	− 0.2	0.1	− 0.1	− 0.3
D_mean_ (%)	0.1	0.1	0.1	0.1
D_1%_ (%)	0.1	0.0	0	0

*GB stent* Stent used in the bile duct,* GI stent* stent used in gastrointestinal tract,* D*_max_ maximum dose,* D*_min_ minimum dose,* D*_mean_:mean dose,* D*_1%_dose of 1% for volume

**Table 2:** Percentage differences in dosimetric parameters according to incident beam angle of MC simulations with GB and GI stents compared to the MC simulations without stent for photon beam

	0°		45°		90°	
	GB stent	GI stent	GB stent	GI stent	GB stent	GI stent
D_max_ (%)	3.9	2.4	0.4	0.0	1.9	1.7
D_min_ (%)	− 1.1	− 1.0	0.2	− 0.1	− 5.9	− 7.0
D_mean_ (%)	0.0	− 0.1	− 0.1	− 0.1	− 1.7	− 0.7
D_1%_ (%)	4.0	2.6	1.4	0.5	0.9	0.9

GB stent; stent used in the bile duct, GI stent: stent used in gastrointestinal tract, D_max_: maximum dose, D_min_: minimum dose, D_mean_: mean dose, D_1%_: dose of 1% for volume

**Table 3:** Percentage differences in dosimetric parameters according to incident beam angle of MC simulations with GB and GI stents compared to the MC simulations without stent for proton beam

	0°		45°		90°	
	GB stent	GI stent	GB stent	GI stent	GB stent	GI stent
D_max_ (%)	0.8	1.1	1.4	1.6	4.4	2.7
D_min_ (%)	− 5.9	− 6.3	− 10.7	− 11.1	− 31.8	− 30.2
D_mean_ (%)	− 0.5	− 0.3	− 0.6	− 0.6	− 4.1	− 2.1
D_1%_ (%)	0.7	0.8	1.0	1.1	3.7	2.1

GB stent; stent used in the bile duct, GI stent: stent used in gastrointestinal tract, D_max_: maximum dose, D_min_: minimum dose, D_mean_: mean dose, D_1%_: dose of 1% for volume

For the proton beam, the values of D_max_ were 0.7 to 4.4% higher than the value without the stent because of the interaction between the proton beam and metal stent. Compared to not using the stent, D_max_ showed the maximum difference (4.4%) for the GB stent located at 90°. The D_min_ showed the maximum difference in dose perturbation value according to the stent from − 5.9 to − 31.8%. The differences in the values of D_min_ were relatively large, − 31.8% and − 30.2% in the GB and GI stents, respectively, for stent locations at 90°. The values of D_mean_ were within 1% of the no-stent values for the GI and GB stents located at 0° and 45° (as in the photon beam), whereas there was a slightly higher difference with the GB and GI stents located at 90°. The comparison of dose using VMAT and double scattering technique was shown to be 0.0–0.5% in Table 4. This result confirms that the dose perturbation results evaluated above are reduced towing to the use of multi beams in clinical cases.

**Table 4:** Percentage difference of dosimetric parameters according to stent position for photon and proton plans in clinical practice

	Photon (VMAT)		Proton (double scattering)	
	GB stent	GI stent	GB stent	GI stent
D_max_ (%)	0.5	0.5	− 0.3	− 0.3
D_min_ (%)	0.2	0.2	− 0.5	− 0.4
D_mean_ (%)	0.0	0.2	− 0.3	− 0.2
D_1%_ (%)	0.1	0.2	− 0.3	− 0.2

GB stent; stent used in the bile duct, GI stent: stent used in gastrointestinal tract, D_max_: maximum dose, D_min_: minimum dose, D_mean_: mean dose, D_1%_: dose of 1% for volume

**Fig. 6:**
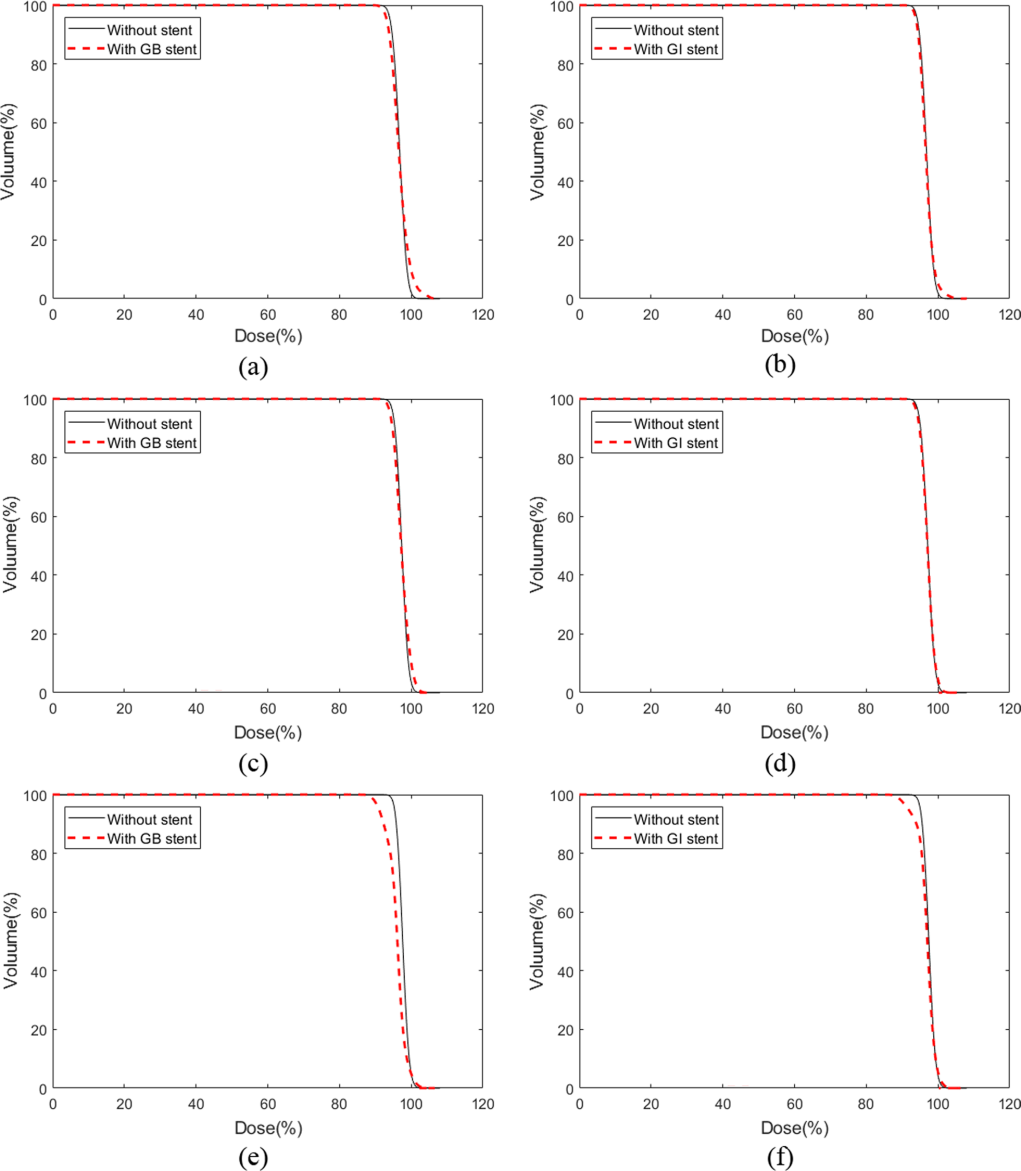
DVH according to the stent’s position and angle compared to no-stent for the photon beam. DVHs calculated from the GB stent **a** and GI stent **b** placed at an angle of 0°, DVHs calculated from the GB stent **c** and GI stent **d** placed at an angle of 45°, and DVHs calculated from the GB stent **e** and GI stent **f** placed at an angle of 90°

**Fig. 7:**
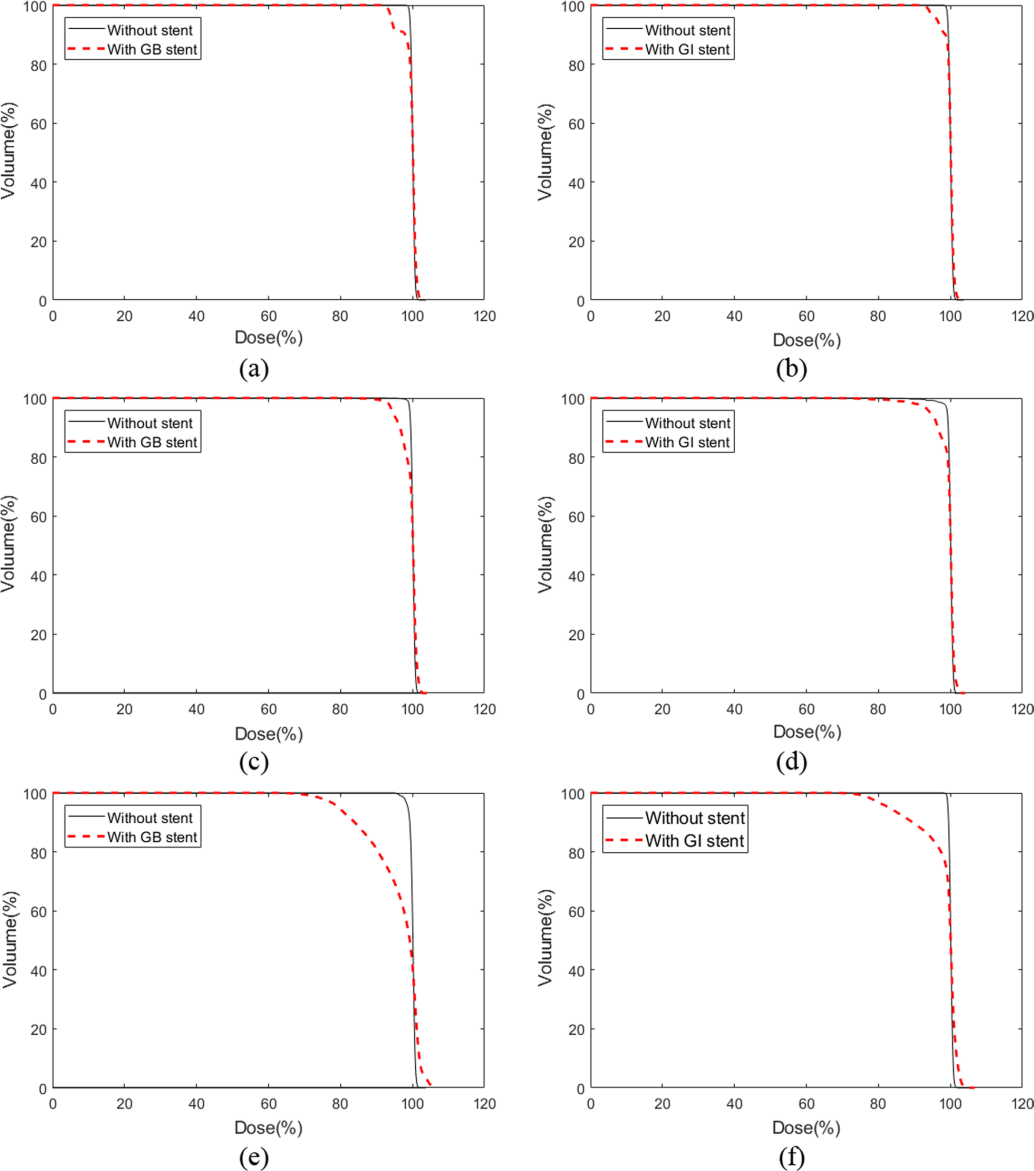
DVH according to the stent’s position and angle compared to no-stent for the proton beam. DVHs calculated from the GB stent **a** and GI stent **b** placed at an angle of 0°, DVHs calculated from the GB stent **c** and GI stent **d** placed at an angle of 45°, and DVHs calculated from the GB stent **e** and GI stent **f** placed at an angle of 90°

Comparisons of the DVHs at various position angles (0°, 45°, and 90°) of the stent with those without the stent are shown in Figs. 6 and 7. The analysis based on the DVHs enables straightforward discrimination of the dose difference with respect to the volume. In both the photon and proton beams, it was observed that the dose varied with the angle of the stent as shown in Figs. 6 and 7.

For the photon beam, the DVHs for the GB and GI stents positioned at 0° showed a difference with respect to the volume of the high-dose region. For the GB and GI stents positioned at 45°, the DVHs were similar to those without the stent. In contrast, the DVHs for the GI and GB stents positioned at 90° showed significant differences, indicating underdoses for the target volume.

For the proton beam, the DVHs indicated underdoses in the target volume for the GI and GB stents at all angles, as compared with the DVHs obtained without stents. The underestimated volume seen in the DVHs was exacerbated as the stent angle was increased. The GI and GB stents positioned at 90° were accompanied by differences in the high-dose region, indicating that high-dosing increased in accordance with the use of the stent. This trend was similar for the photon and proton beams; however, the difference was significantly larger for the proton beam than for the photon beam.

## Discussion

Film dosimetry is often used to evaluate the effect of radiation distribution by artefacts during RT [7]. However, it is difficult to three-dimensionally evaluate the dose distribution for the proton beam because of the energy dependence of the film. Various measuring methods such as a thermoluminescent dosimeter (TLD), optically stimulated luminescent dosimeter (OSLD), and chamber are widely used to measure dose perturbation, but these methods also have limitations in three-dimension measurement. Because the stent causes artefacts and blurring in a CT image, the areas where these blemishes occur are usually assigned with the Hounsfield unit (HU) value of the surrounding tissue for treatment planning. Previous studies using film and TLD measurements have reported that these blemishes indicate dose differences. There are studies reportinged that the body is composed of substances with different densities, which can result in dose changes of up to 20% [24, 25]. In this study, dose fluctuations caused by metal stents used in clinical practice for photon beams and proton beams were evaluated through MC simulations. The MC simulations accounted for most of the physical processes leading to dose deposition, including mean energy loss, range straggling, multiple coulomb scattering, and nuclear reactions.

To perform an appropriate simulation using TPS and MC, a metal stent was inserted into the intensity modulated radiation therapy (IMRT) phantom, and CT scan was performed with the phantom. The wire thickness of the used stent was 0.1 mm, which was smaller than the size of the CT detector. Consequently, the stent did not appear clearly on the CT image, producing a blurry image instead. The stent density was assigned an average value corresponding to the densities of the surrounding materials, which amounted to 1.08–1.3 g/cm^3^, a range of densities considerably lower than the actual density of the stent [10]. Therefore, the dose variations calculated from the TPS with the inclusion of metal stents showed little difference between the photon beam and proton beam. The authors considered these calculated values to be inaccurate representations of reality, and we conducted an MC simulation to verify the differences in dose variation.

To analyse the dosimetric parameters such as D_max_, D_mean_, D_min_, and D_1%_, the dose enhancement was observed in D_max_ because of the multiple Coulomb scattering caused by the interactions with the high-density material. Compared to the no-stent case, the maximum difference in D_max_ was found when the stent was positioned at 0° for the photon beam and at 90° for the proton beam. Values of D_min_ were lower for both the GB and the GI stents positioned at all angles, except for the stents positioned at 45°. The value of D_min_ indicated that the degree of underdose increased as the angle of the positioned stent increased. In addition, the differences observed for the proton beam were more pronounced than those observed for the photon beam. Both the photon and proton beams showed a more significant reduction when the GB and GI stents were positioned at 90° than when they were positioned at 0° and 45°. This was because both the photon and proton beams interacted more intensively with the stent when they were incident on the stents positioned at 90°, due to the overlapping effect. However, it is expected that the differences observed in this study will not be replicated in the clinical situation because the positioning of a stent at 90° is rarely applied. In the MC simulation for clinical practice, very small dose differences were observed. The values of D_mean_ for both the photon and proton beams also indicated reduced doses for the stents located at all angles, compared with those obtained without a stent.

The analysis of the DVHs indicated that there was an underdose to the target volume when the stent was positioned at the specified angles, as compared with the no-stent dose, for both the photon and proton beams. The largest dose reduction in the DVHs occurred with the stent located at 90°, for both the photon and proton beams. In addition, the proton beam displayed a greater dose reduction for the target volume than the photon beam. This may have occurred because of the influence of the dose shadow on the proton beam [8]. A comparison of Figs. 4 and 5 reveals that the proton beam had an underdose in the black colour around the stent, as compared with the photon beam. The calculated results of the MC simulation were evaluated with a statistical uncertainty that lay within 1% for all voxels.

This study has two limitations. One limitation is that the stent modelling for the MC calculation consisted of only nitinol wire. However, the tip of the nitinol wire attached to the stent was gold. Because this part occupies a relatively small proportion of the entire stent composition, it was excluded from the stent modelling for MC simulation. In this gold portion of the stent, the effect of the dose difference on the overall volume was very small. However, there was a 5% difference for the point dose in the MC simulation. In future studies, a simulation using accurate stent modelling which includes the complete composition of the stent will lead to a more accurate dose analysis. The second limitation of the study is that dose perturbations by the stent for the proton and photon plans were evaluated through simulations based on a single fraction with AP/PA fields, VMAT, and double scattering technique. Treatment plans used in actual clinical practice are complex creations with various beam numbers and incident beam angles. However, the dosimetric results of this study obtained under few conditions may have shown a relatively large difference. Therefore, it is expected that clinical plans that can use a variety of beam incidence direction and numbers will produce reduced results rather than differences of our results obtained in this study. To obtain more accurate results, we will include more patient data and different treatment technique in our future studies of proton beams and photon beams which include the use of stents.

## Conclusions

This study confirmed that there was a difference in the dose delivered within the target volume by using MC simulation, which could not confirm the dose perturbation in the target volume even in the presence of the metal stent in the TPS calculation. Through MC simulations of photon and proton plans, we demonstrated that there are differences in the dosimetric parameters of the target volume as compared with those without a stent, for the positions and angles of the stent inserted into the phantom. Furthermore, we found that the stent caused a dose reduction rather than a dose increase of the target volume. This phenomenon appeared more clearly in the proton beam than in the photon beam.

Based on the results obtained from the MC simulation, we believe that delivery of the treatment beam avoiding the stent is the best method to prevent underdose and overdose in the target volume. However, we recommend that it is important to reduce the effect by using as many fractions and beams as possible, to reduce the effect of the stent when the beam is delivered.

## Data Availability

The datasets supporting the study conclusions are included within this manuscript.

## Abbreviations

TPS: Treatment planning system
MC: Monte Carlo
AP: Anterior–posterior
PA: Posterior–anterior
DVH: Dose volume histogram
HCC: Hepatocellular carcinoma
RT: Radiotherapy
VMAT: Volumetric modulated arc therapy
PBT: Proton beam therapy
High-Z: High atomic number
SEMS: Self-expanding metal stents
ROI: Region of interest
CT: Computed tomography
TLD: Thermoluminescent dosimeter
OSLD: Optically stimulated luminescent dosimeter
IAEA: International atomic energy agency
D_max_: Maximum dose
D_min_: Minimum dose
D_mean_: Mean dose
DICOM: Digital imaging and communications in medicine
HU: Hounsfield unit
